# A modified anthrax toxin-based enzyme-linked immunospot assay reveals robust T cell responses in symptomatic and asymptomatic Ebola virus exposed individuals

**DOI:** 10.1371/journal.pntd.0006530

**Published:** 2018-05-24

**Authors:** Bobby Brooke Herrera, Donald J. Hamel, Philip Oshun, Rolake Akinsola, Alani S. Akanmu, Charlotte A. Chang, Philomena Eromon, Onikepe Folarin, Kayode T. Adeyemi, Christian T. Happi, Yichen Lu, Folasade Ogunsola, Phyllis J. Kanki

**Affiliations:** 1 Department of Immunology and Infectious Diseases, Harvard T.H. Chan School of Public Health, Boston, MA, United States of America; 2 College of Medicine, University of Lagos, Akoka, Lagos, Nigeria; 3 Lagos University Teaching Hospital, Idi Oro, Lagos, Nigeria; 4 African Centre of Excellence for Genomics of Infectious Diseases (ACEGID), Redeemer’s University, Ede, Osun State, Nigeria; 5 Department of Biological Sciences, College of Natural Sciences, Redeemer’s University, Ede, Osun State, Nigeria; 6 Haikou VTI Biological Institute, Haikou, Hainan, China; University of Geneva Hospitals, SWITZERLAND

## Abstract

**Background:**

Ebola virus (EBOV) caused more than 11,000 deaths during the 2013–2016 epidemic in West Africa without approved vaccines or immunotherapeutics. Despite its high lethality in some individuals, EBOV infection can produce little to no symptoms in others. A better understanding of the immune responses in individuals who experienced minimally symptomatic and asymptomatic infection could aid the development of more effective vaccines and antivirals against EBOV and related filoviruses.

**Methodology/Principle findings:**

Between August and November 2017, blood samples were collected from 19 study participants in Lagos, Nigeria, including 3 Ebola virus disease (EVD) survivors, 10 individuals with documented close contact with symptomatic EVD patients, and 6 control healthcare workers for a cross-sectional serosurvey and T cell analysis. The Lagos samples, as well as archived serum collected from healthy individuals living in surrounding areas of the 1976 Democratic Republic of Congo (DRC) epidemic, were tested for EBOV IgG using commercial enzyme-linked immunosorbent assays (ELISAs) and Western blots. We detected antibodies in 3 out of 3 Lagos survivors and identified 2 seropositive individuals not known to have ever been infected. Of the DRC samples tested, we detected antibodies in 9 out of 71 (12.7%). To characterize the T cell responses in the Lagos samples, we developed an anthrax toxin-based enzyme-linked immunospot (ELISPOT) assay. The seropositive asymptomatic individuals had T cell responses against EBOV nucleoprotein, matrix protein, and glycoprotein 1 that were stronger in magnitude compared to the survivors.

**Conclusion/Significance:**

Our data provide further evidence of EBOV exposure in individuals without EVD-like illness and, for the first time, demonstrate that these individuals have T cell responses that are stronger in magnitude compared to severe cases. These findings suggest that T cell immunity may protect against severe EVD, which has important implications for vaccine development.

## Author summary

The 2013–2016 West African Ebola virus (EBOV) outbreak is the largest on record with over 28,000 reported symptomatic cases and more than 11,000 deaths. We developed a simple and inexpensive modified anthrax toxin-based ELISPOT assay to detect and characterize the T cell responses elicited by prior exposure to EBOV. Our data show robust T cell responses to several EBOV proteins in individuals who experienced both severe and asymptomatic EBOV infections. These results provide further evidence that EBOV transmission events can go undetected. We also show that the seropositive asymptomatic individuals have stronger T cell responses compared to survivors, which has important implications for vaccine development.

## Introduction

Ebola viruses including the five antigenically distinct species, *Bundibugyo ebolavirus* (BDBV), *Reston ebolavirus* (RESTV), *Tai Forest ebolavirus* (TAFV), *Sudan ebolavirus* (SUDV), and *Zaire ebolavirus* (EBOV), are enveloped filamentous viruses belonging to the family *Filoviridae* [1–3]. These viruses carry negative strand RNA genomes approximately 19 kb in length that code for 7 structural proteins: the nucleoprotein (NP), VP35, the matrix protein (VP40), the glycoprotein (GP), VP30, VP24, and the RNA-dependent RNA polymerase [1].

Since the discovery of EBOV in 1976 in the Democratic Republic of Congo (DRC), BDBV, SUDV, and EBOV have caused sporadic epidemics of lethal hemorrhagic fever or Ebola virus disease (EVD), largely in Central Africa, with case fatality rates of 23–90% [4–7]. In December 2013, an outbreak of EBOV was detected in Guinea, which led to the largest ever recorded epidemic spanning seven West African countries. By 2016, more than 28,000 symptomatic cases of EVD were reported with a case fatality rate of 40% [8]. Although there are several in the pipeline, there are currently no licensed vaccines against EBOV and treatment remains largely supportive.

Richardson et al. recently conducted a survey of close household contacts of individuals who had severe EVD and showed that a significant portion of EBOV transmission events went undetected during the West African outbreak because some individuals contracted infection but had mild illness or were asymptomatic [9]. This work adds to a growing body of evidence suggesting that despite its high lethality in some individuals, EBOV infection can produce little to no symptoms in others [10]. Seroprevalence surveys conducted in Africa have historically described asymptomatic EBOV infection, however, due to uncertainty in serologic assays, consensus on the significance of these findings has not been reached. Nonetheless, while several hypotheses might explain minimally symptomatic and asymptomatic EBOV infection, including properties of the infecting virus (e.g. less virulent isolate), low inoculum, route of transmission, host factors (e.g. resistance through viral cell receptor polymorphism), or a robust innate and/or adaptive immune response are potential explanations.

Antibody therapy is considered an effective and powerful treatment strategy against many infectious pathogens. While studies of whole-blood transfusion or serum as passive immunity for EBOV treatment have demonstrated limited efficacy, monoclonal antibodies have shown promise in animal models and have been tested in human clinical trials [11–15]. The most successful of these is the antibody cocktail ZMapp, which comprises three humanized murine monoclonal antibodies that target the EBOV GP. ZMapp reversed advanced disease and rescued 100% of rhesus macaques up to 5 days post-viral challenge; however, it did not meet a pre-specified threshold for efficacy in humans when tested during the 2013–2016 outbreak [13, 16].

Immune depletion studies in the macaque EBOV model demonstrated that humoral responses were beneficial in containing virus, but CD8+ T cells were essential for vaccine-induced protection. These findings suggest that humoral immunity alone may not account for full recovery or secondary protection. T cell responses of appropriate quality and magnitude were shown to be important for human protection against EBOV [17–19]. During the West African outbreak, Ruibal et al. demonstrated unique mechanisms that regulate T cell homeostasis in fatal and non-fatal EVD cases, suggesting that EBOV infections can trigger T cell responses that may be more effective in some individuals than others [20]. More studies are needed to better understand the immunopathogenesis of EVD, especially in mildly symptomatic or asymptomatic cases.

In this study, we adapted the modified anthrax lethal factor (LFn) delivery system to enable the detection and characterization of T cell responses in previously EBOV exposed individuals from Lagos, Nigeria, three years after the outbreak. We fused the EBOV (Makona variant) NP, VP40, and the receptor binding subunit of GP, known as GP1, to LFn. The LFn-EBOV recombinant proteins were expressed and used as antigens to stimulate peripheral blood mononuclear cells (PBMCs) in an ELISPOT assay. We report robust T cell responses in EVD survivors and in EBOV seropositive asymptomatic individuals.

## Methods

### Ethics statement

The protocols used in this study were approved by the Health Research Ethics Committee at the College of Medicine, University of Lagos, Nigeria (CM/HREC/09/16/055) and by the Internal Review Board (IRB) at the Harvard T.H. Chan School of Public Health (Harvard Chan), Boston, USA (Protocol number IRB16-1321). All study participants included in the study were adults. The participants from Lagos provided written informed consent for the collection of samples and corresponding data were banked and de-identified prior to analyses. The DRC samples were collected under surveillance in 1976. The samples were anonymized by the US CDC prior to shipment to Harvard Chan in 1985.

### Study populations

On 20 July 2014 an individual who travelled from Liberia presenting with fever was transported to a private hospital in Lagos; he denied contact with known EVD cases and was treated with antimalarial drugs [21]. The individual’s condition worsened over the next three days, at which point EVD was suspected. Filovirus and EBOV-specific PCR testing was performed by Lagos University Teaching Hospital (LUTH) and Redeemer’s University (RUN) African Centre of Excellence for Genomics of Infectious Diseases (ACEGID), respectively, and the patient was confirmed EVD positive on 25 July 2014; he died on that day. During this period, many were exposed to the virus and all contacts were traced, placed under surveillance, and monitored for clinical features of EVD. The chain of EVD transmission from this single individual resulted in 19 laboratory-confirmed cases, 8 of whom succumbed to infection. Nigeria was declared EBOV free by the World Health Organization on 20 October 2014.

Between August and November 2017, we recruited 3 EVD survivors with RT-PCR positive results for EBOV, 10 individuals with documented close contact with symptomatic EVD patients, who remained healthy during the outbreak, and 6 healthcare workers (HCWs) from a different hospital in Lagos with a low likelihood of previous EBOV exposure based on proximity to the outbreak epicenter and subsequent questionnaires to eliminate potential recall bias. All methods described subsequently were performed on the Lagos samples from the EVD survivors, documented EVD contacts, and controls.

Additionally, an epidemic of EVD erupted in Yambuku, a small village near Yandongi, Democratic Republic of Congo (formerly Zaire) in 1976 [4]. In the course of epidemiological investigations of this outbreak, hundreds of serum samples were collected from residents of the surrounding areas. In 1985, these serum samples were screened for antibodies to human immunodeficiency virus (HIV) [22], and in 1988 additional screening was conducted at Harvard Chan, with excess samples stored. The availability of 71 archived samples from the first known EBOV outbreak offered an opportunity to assess EBOV-specific antibodies in serum collected from potentially EBOV exposed but presumed uninfected individuals. Only Western blot testing to EBOV fusion antigens was performed on the DRC samples for EBOV serostatus.

### PBMC and plasma isolation performed at LUTH

PBMCs were separated from plasma and whole blood in EDTA tubes by Ficoll-Paque gradient density (GE Healthcare Life Sciences, Pittsburgh, PA, USA) and cryopreserved in freezing media (10% dimethyl sulfoxide [DMSO, Sigma-Aldrich, St. Louis, MO, USA], 90% fetal bovine serum [FBS, ThermoFisher Scientific, Rockford, IL, USA) at -80°C prior to transfer to liquid nitrogen. Plasma was separately aliquoted and immediately transferred to -80°C.

### Nucleic acid testing

RNA was extracted from all plasma samples using the QIAamp RNA Viral Kit (Qiagen, Hilden, Germany) according to the manufacturer’s instructions and was quantified using quantitative RT-PCR (qRT-PCR) for both EBOV and human ribosomal RNA (18S), as previously described [23]. Briefly, the assay mix included 3ul of RNA, 0.03umol/L sense and anti-sense primers, 5ul of X2 Power ZYBR Green RT-PCR Mix and 0.08ul of RT Enzyme Mix (ThermoFisher Scientific, Rockford, IL, USA). The cycling conditions were 48°C for 30 min, 95°C for 10 min, followed by 45 cycles of 95°C for 15 sec, 60°C for 30 sec with a melt curve of 95°C for 15 sec, 55°C for 15 sec, 95°C for 15 sec. qRT-PCR was performed on the LightCycler 96 (Roche, Indianapolis, IN, USA). The amplicons were cleaned using AMPure XP beads (Beckman Coulter, Brea, CA, USA) according to the manufacturer’s instructions and amplicon concentrations were converted to EBOV copies per microliter for quantification.

### Antigen and antibody ELISAs

All plasma samples collected from the Lagos study participants were screened by the ReEBOV IgG ELISA kit (Zalgen Lab, Germantown, MD, USA) and the EV-IgG ELISA kit (MyBioSource, San Diego, CA, USA) according to the manufacturers’ instructions. All Lagos study participants’ plasma were also screened by the ReEBOV Antigen ELISA kit (Zalgen Lab, Germantown, MD, USA) according to the manufacturer’s instructions. Measurement of OD was performed within 5 min of stopping the reaction at 450nm (antibody) and 620nm (antigen) with the MiltiskanTM GO Plate Reader (ThermoFisher Scientific, Rockford, IL, USA).

### Construction and expression of LFn fusion proteins

Commercially synthesized amino acid fragments corresponding to the EBOV (Makona variant) NP, VP40, GP1, and sGP were cloned into the LFn expression plasmid (pET15bLFn), which contains a T7 promoter, histidine tag, and the terminal domain of the anthrax lethal factor. The EBOV NP, VP40, GP1, and sGP sequences were derived consensus sequences generated from the initial 99 genomes initially published during the 2013–2016 outbreak [24]. The pET15bLFn containing the coding sequences of EBOV NP, VP40, and GP1 were transformed into E. coli BLR (DE3) (Millipore, Medford, MA, USA) for expression. Clones containing the correct reading frame as verified by sequencing were used for protein expression, as previously described [25]. The pET15bLFn was expressed and purified as described above for use as a negative control.

### Western blot

All plasma samples collected from both the Lagos and DRC study participants were screened for the presence of IgG antibodies to bind to recombinant LFn-EBOV-GP1 and -sGP. Briefly, 50ug LFn-EBOV-GP1 and -sGP were added to reducing buffer (2% SDS, 0.5 M Tris [pH 6.8], 20% glycerol, 0.001% bromophenol blue, and 5% 2-Mercaptoethanol) and subjected to 12% polyacrylamide gel electrophoresis (PAGE). Western Blot analysis was conducted using plasma samples (1:100) as primary antibody and anti-human IgG HRP (ThermoFisher Scientific, Rockford, IL, USA) as secondary antibody. Visualization was performed using SuperSignal Femto Chemiluminescent Substrate (ThermoFisher Scientific, Rockford, IL, USA) and with the Chemi Doc XRS+ Imaging System (Bio Rad Technologies, Hercules, CA, USA).

### Ex vivo ELISPOT assay

ELISPOT assays were performed as previously described [26]. Briefly, a total of 2x10^5^ PBMCs were incubated with 0.1ml complete RPMI 1640 medium (ThermoFisher Scientific, Rockford, IL, USA) in the presence of 2.5ug/ml final concentration LFn fusion proteins in antibody coated 96-well polyvinylidene difluoride (PVDF)-backed MultiScreen_HTS_ (MSIP) microtiter plates (Millipore, Medford, MA, USA). After 24 hr of incubation at 37°C, the plates were washed and incubated with secondary antibodies, followed by an overnight incubation at 4°C. Plates were washed, then incubated for 2 hr at room temperature with the enzymatic conjugate. Spots were developed using Vector Blue substrate solution (Vector Laboratories, Burlingame, CA, USA) and counted manually using a stereozoom microscope (20X magnification). In CD8 and CD4 experiments, CD8+ and CD4+ T cells were isolated from PBMCs using the CD8+ and CD4+ T cell Isolation kits (Miltenyi Biotec, Auburn, CA, USA). EBOV-specific spots were calculated by subtracting the mean of the negative control values of the replicates from the mean values of the specific stimulation. Positive responses were greater than four times the mean background, three standard deviations above the background, and ≥55 spot-forming cells per (SFC)/10^6^ PBMCs.

### Statistical analysis

Statistical analyses, including comparison between groups of individuals and between antigens, were performed using Prism 7 (GraphPad, San Diego, CA, USA). Data are expressed as geometric positive means ± standard deviation. Non-parametric Mann-Whitney test was used to compare data and determine statistical significance. p<0.05 was considered significant.

## Results

### Antigen capture, qRT-PCR, and serology data

At the time of sample collection, all Lagos study participants recruited were healthy. The median age was 34 years (range, 24–65 years), and 37% were female. Study participant characteristics are summarized in S1 Table.

**Fig 1:**
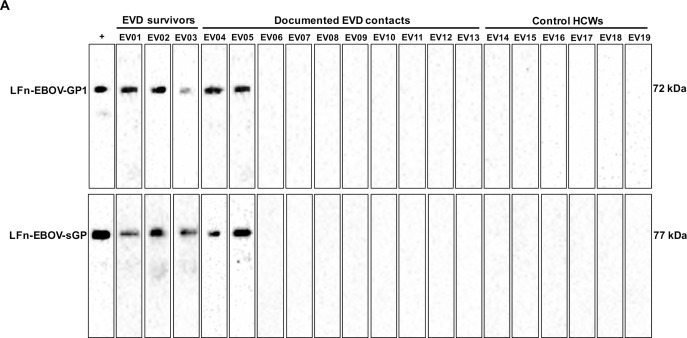
Detection of EBOV antibodies, Lagos, Nigeria. (A) Sera samples from 3 EVD survivors, 10 documented EVD contacts, and 6 control HCWs were subjected to LFn-EBOV-GP1 and LFn-EBOV-sGP Western blot analysis. +, positive control. Molecular size marker units are kDa. EVD, Ebola virus disease. Control HCWs, control healthcare workers.

We assessed Lagos plasma samples for the presence of EBOV nucleic acid, EBOV antigen, and EBOV-specific antibodies (Table 1). All study participants tested negative for EBOV by qRT-PCR and antigen capture ELISA. Using the Zalgen ReEBOV IgG ELISA, which assesses antibodies specific to EBOV VP40, 3 of 3 EVD survivors and 2 of 10 documented EVD contacts tested positive, and all 6 control HCWs were negative. Using the MyBioSource EV-IgG ELISA, which uses inactivated and homogenized EBOV, 2 of 3 EVD survivors and the same 2 out of 10 EVD contacts tested positive while the control HCWs remained negative. Consistent with the Zalgen ReEBOV IgG ELISA, Western blot analysis using the LFn-EBOV-GP1 and -sGP fusion proteins revealed GP1- and sGP-specific IgG antibodies in the 3 EVD survivors and the same 2 out of 10 documented EVD contacts, with no detectable antibodies in the control HCWs (Fig 1).

**Table 1:** qRT-PCR, antigen ELISA, and serology results, Lagos, Nigeria.

Study participants	qRT-PCR	ReEBOV Antigen ELISA	ReEBOV IgG ELISA	EV-IgG ELISA	LFn-EBOV-GP1 Western blot	LFn-EBOV-sGP Western blot
EVD survivors	0/3	0/3	3/3	2/3	3/3	3/3
Documented EVD contacts	0/10	0/10	2/10	2/10	2/10	2/10
Control healthcare workers	0/6	0/6	0/6	0/6	0/6	0/6

**Fig 2:**
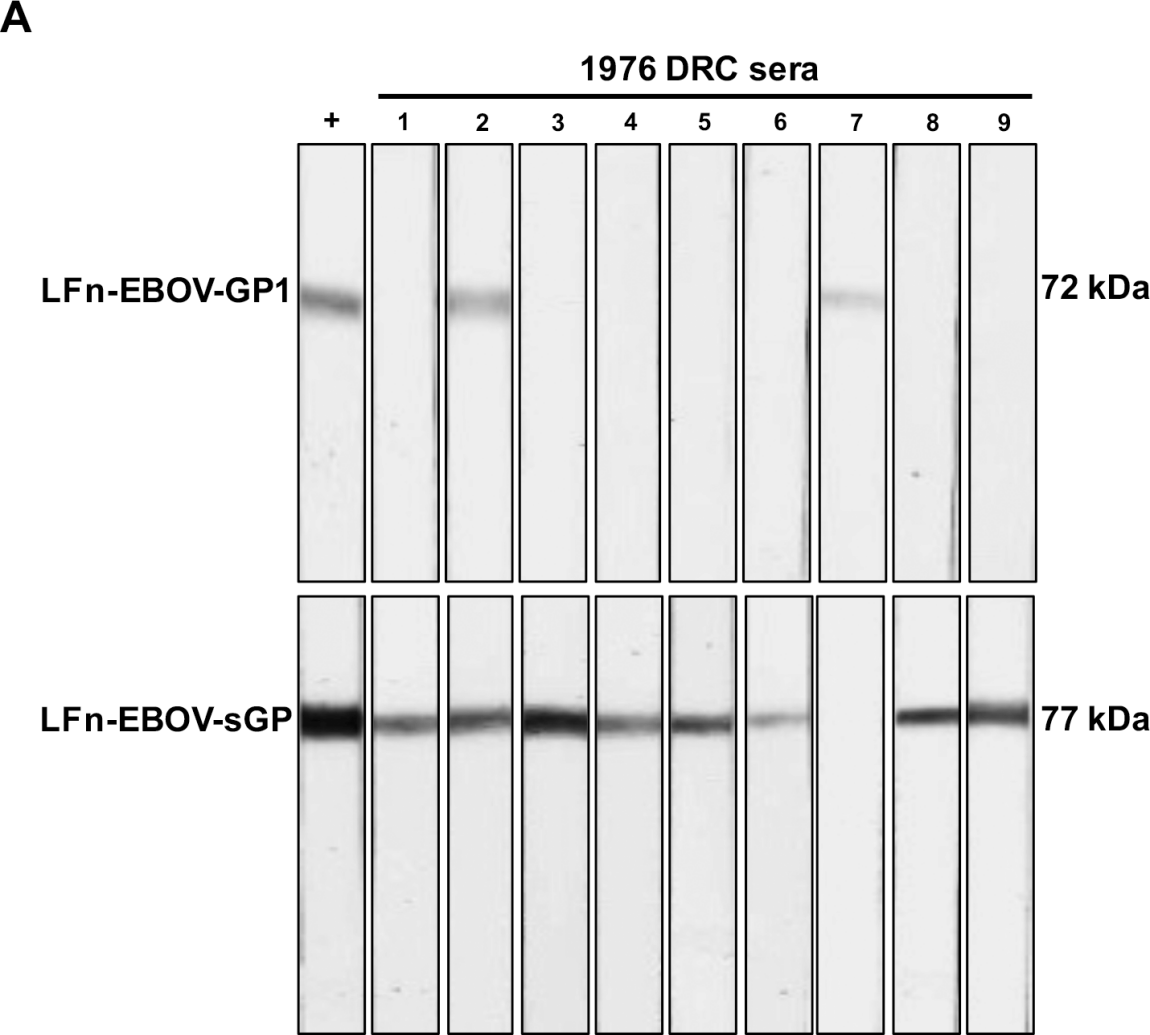
Detection of EBOV antibodies, 1976 Democratic Republic of Congo. (A) Representative image of reactive LFn-EBOV-GP and LFn-EBOV-sGP Western blot analysis in 9 sera collected in surrounding areas of the 1976 Ebola virus outbreak in the Democratic Republic of Congo. +, positive control. Molecular size marker units are kDa. EVD, Ebola virus disease.

Of the serum samples collected during the 1976 EVD epidemic in the DRC, 9 of 71 (12.7%) contained IgG antibodies that were reactive to LFn-EBOV-GP1 and/or -sGP as demonstrated by Western blot (Table 2, Fig 2). Antibodies specific to LFn-EBOV-GP1 were found in 1.4% (1/71), 9.6% (7/71) to LFn-EBOV-sGP and 1.4% (1/71) to both LFn-EBOV-GP1 and -sGP.

**Table 2:** Serology results, 1976 Democratic Republic of Congo.

1976 DRC sera (N = 71)	LFn-EBOV-GP1 Western blot	LFn-EBOV-sGP Western blot	LFn-EBOV-GP1 + -sGP Western blot
No. positive/Total number tested (%)	1/71 (1.4)	1/71 (1.4)	9/71 (12.7)

### *Ex vivo* ELISPOT data

**Fig 3:**
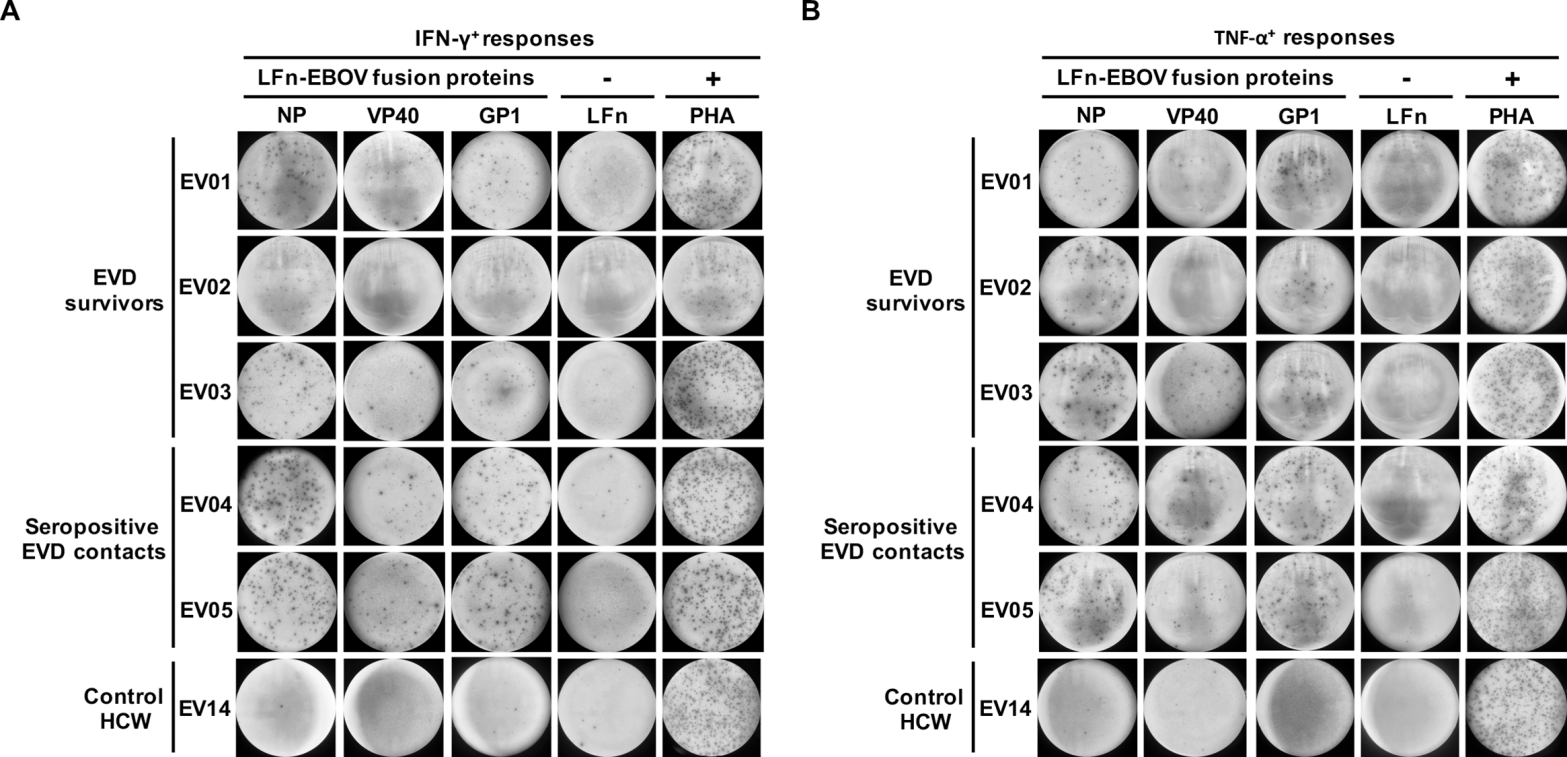
Cellular immune responses, Lagos, Nigeria. PBMC samples from the EVD survivors, documented EVD contacts, and control HCWs were treated with the LFn-EBOV fusion proteins and the IFN-γ+ and TNF-α+ cellular responses were detected by LFn ELISPOT ex vivo experiments. Representative image of IFN-γ (A) and TNF-α (B) cellular responses when stimulated with the LFn-Ebola virus fusion proteins. NP, LFn-EBOV-NP. VP40, LFn-EBOV-VP40. GP1, LFn-EBOV-GP1. LFn, negative control. PHA, phytohemaglutanin, positive control.

We then assessed the post-infection cellular responses in all Lagos study participants by LFn fusion protein stimulation of PBMCs in IFN-γ and TNF-α ELISPOTs. All 3 EVD survivors and the 2 seropositive EVD contacts mounted detectable IFN-γ and TNF-α cellular responses to LFn-EBOV-NP, -VP40, and/or -GP1 (Table 3, Fig 3). The remaining 8 documented EVD contacts and all 6 control HCWs mounted cellular responses below the positive threshold (Table 3).

**Table 3:** Ex vivo ELISPOT results using the LFn-EBOV fusion proteins, Lagos, Nigeria.

Study participants	EBOV seropositive	IFN-γ^+^ SFC / 10^6^ PBMC			TNF-α^+^ SFC / 10^6^ PBMC		
		LFn EBOV NP	LFn EBOV VP40	LFn EBOV GP1	LFn EBOV NP	LFn EBOV VP40	LFn EBOV GP1
**EVD survivors**							
EV01	Y	572	187	400	522	84	440
EV02	Y	418	30	366	455	29	120
EV03	Y	508	75	313	337	120	383
**Documented EVD contacts**							
EV04	Y	705	135	552	605	165	498
EV05	Y	770	250	590	670	214	562
EV06	N	12	9	14	5	12	13
EV07	N	23	17	18	10	15	16
EV08	N	7	9	13	7	9	8
EV09	N	33	20	18	8	4	7
EV10	N	6	10	8	8	9	11
EV11	N	2	12	7	15	14	18
EV12	N	14	9	13	4	6	12
EV13	N	10	12	6	8	12	11
**Controls healthcare workers**							
EV14	N	8	3	12	5	11	7
EV15	N	16	13	23	9	10	6
EV16	N	27	14	19	12	6	17
EV17	N	16	7	9	5	10	11
EV18	N	3	8	13	4	7	12
EV19	N	12	21	16	14	9	8

**Fig 4:**
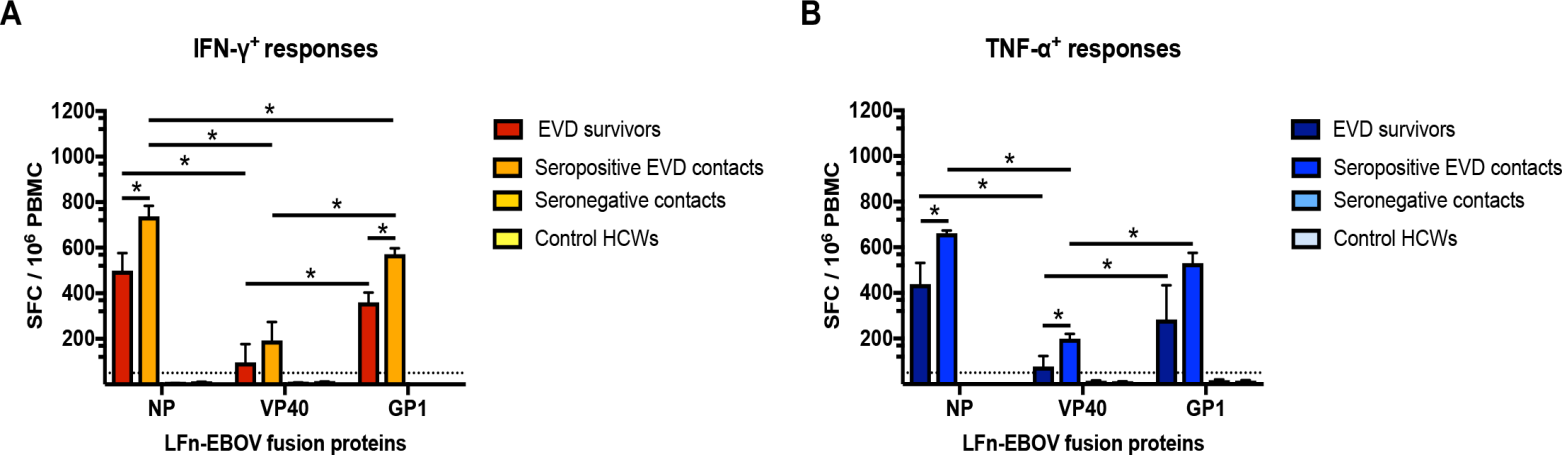
Ex vivo cellular reactivity to EBOV LFn fusion proteins, Lagos, Nigeria. PBMC samples from the EVD survivors and documented EVD contacts were treated with the LFn-EBOV fusion proteins and the IFN-γ+ and TNF-α+ cellular responses were detected by LFn ELISPOT ex vivo experiments. Average magnitude of convalescent IFN-γ (A) and TNF-α (B) responses are shown. Dotted lines represent the cut-off value. Control HCWs, control healthcare workers. *, p < 0.05.

In most cases, the EVD survivors and the seropositive contacts mounted the strongest IFN-γ and TNF-α cellular against LFn-EBOV-NP, p<0.05. (Fig 4). IFN-γ and TNF-α cellular responses against LFn-EBOV-GP1 were stronger compared to responses against LFn-EBOV-VP40, p<0.05. Additionally, the seropositive EVD contacts mounted IFN-γ and TNF-α cellular responses against LFn-EBOV-NP, -VP40, and -GP1 that were stronger in magnitude compared to survivors; similarly, IFN-γ responses against LFn-EBOV-NP and -GP1 and for TNF-α responses against LFn-EBOV-NP and -VP40 were stronger in contacts compared to survivors, p<0.05.

**Fig 5:**
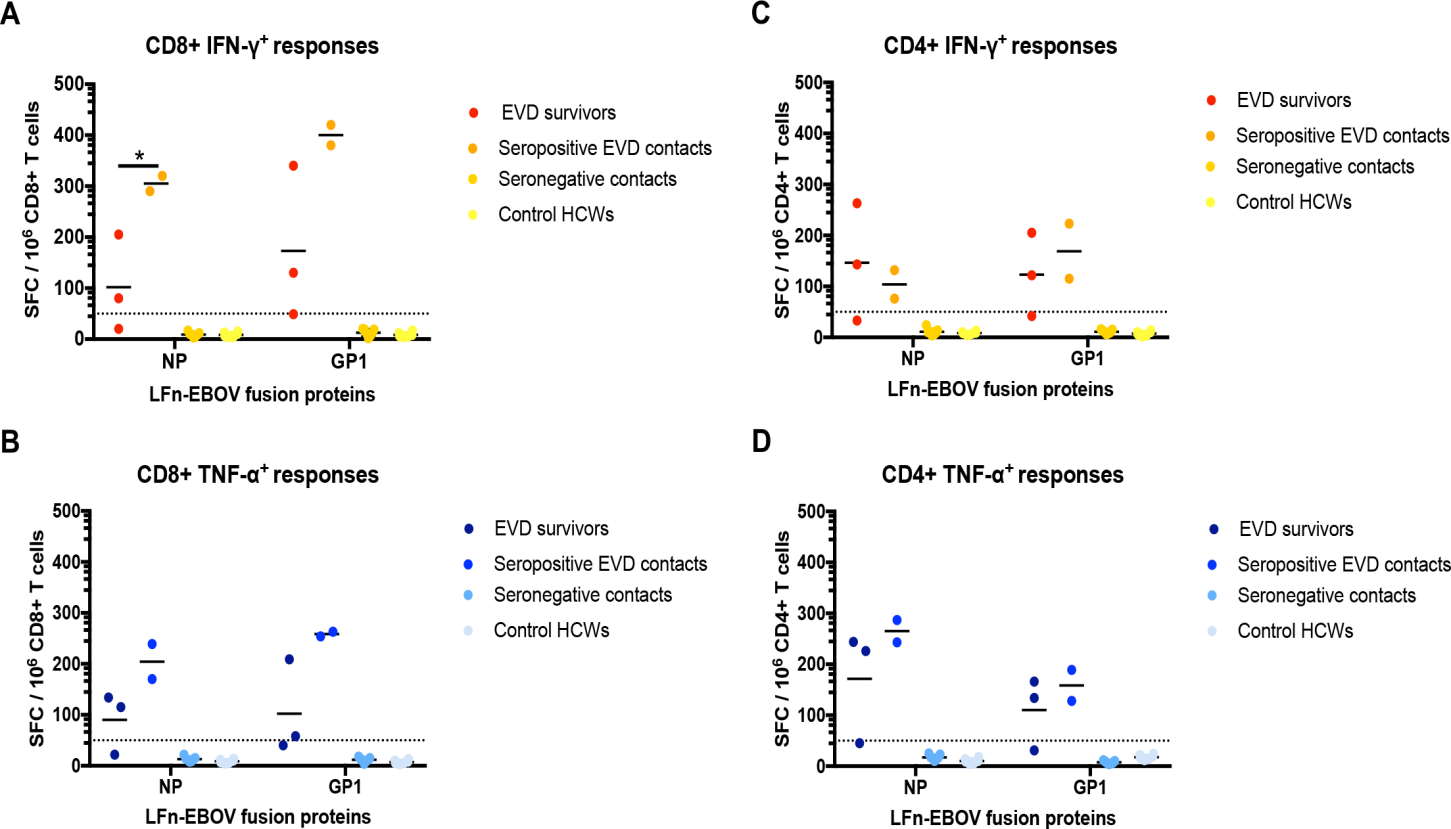
Ex vivo CD8+ and CD4+ cellular reactivity to EBOV LFn fusion proteins, Lagos, Nigeria. CD8+ and CD4+ cells from the EVD survivors and documented EVD contacts were treated with the LFn-EBOV fusion proteins and the IFN-γ+ and TNF-α+ responses were detected by LFn ELISPOT ex vivo experiments. Individual and mean CD8+ IFN-γ+ (A) and TNF-α+ (B) and CD4+ IFN-γ+ (C) and TNF-α+ (D) responses are shown. Dotted lines represent the cut-off value. Control HCWs, control healthcare workers. *, p<0.05.

We further evaluated the post-infection CD8+ and CD4+ T cell responses in the EVD survivors and the seropositive EVD contacts. Due to sample availability, we assessed IFN-γ and TNF-α responses against LFn-EBOV-NP and -GP1. In nearly all cases, mean IFN-γ and TNF-α CD8+ and CD4+ responses were stronger among the seropositive EVD contacts compared to the survivors, though not statistically significant (Fig 5). As the only exception, mean IFN-γ CD4+ response against LFn-EBOV-NP was stronger in the EVD survivors compared to the seropositive contacts, though not statistically significant (Fig 5C).

## Discussion

The characterization of the immune response in mild and asymptomatic EBOV infection may explain how certain individuals infected with this otherwise highly lethal virus avoid severe disease. This could contribute to efforts in the development of more effective vaccines and immunotherapeutics against EBOV and related filoviruses. Therefore, we adapted the modified anthrax toxin delivery system to design LFn-EBOV fusion proteins for antibody and/or T cell analyses in EVD survivors, documented close EVD contacts, and in presumed healthy individuals living in nearby areas of the 1976 EVD outbreak in the DRC.

T cell responses in survivors of EVD epidemics has been relatively well studied. Four patients with acute EVD demonstrated CD8+ and CD4+ T cell activation against several viral proteins, and this activation persisted for up to one month after infection [17]. Consistent with this study, prolonged T cell activation was observed in a single patient who cleared EBOV infection without the use of experimental drugs [27]. Another study evaluating patients during the acute phase of infection, demonstrated that survivors have T cells that express lower levels of the inhibitory molecules CTLA-4 and PD-1, compared to fatal EVD cases with high viral load [20]. Additionally, a study of SUDV survivors, 12 years post infection, demonstrated strong memory CD4+, but not CD8+, T cell activation and neutralizing humoral immunity [28]. Whole irradiated SUDV was used to stimulate PBMC samples in these long-recovered survivors, likely contributing to the limited CD8+ activation.

Moreover, minimally symptomatic and asymptomatic EBOV infections are not new phenomena. Using an immunofluorescence assay, World Health Organization researchers identified EBOV-infected individuals who had symptoms that ranged in severity, from mild to rapidly fatal, during the first outbreaks of EBOV in Zaire and Sudan in 1976 and 1979, respectively [4, 5, 29]. Since then, a number of additional studies utilizing diverse methods have identified EBOV-infected individuals who nonetheless remained asymptomatic [30–34]. However, studies examining the cellular immune responses during mild or asymptomatic infection are scarce. In one study of Gabonese individuals with asymptomatic EVD infection, high concentrations of pro-inflammatory cytokines were detected in plasma samples; yet, no T cell-derived cytokines were observed [33]. A follow-up study in the same individuals demonstrated mRNA expression of T cell cytokines and cytotoxic activation markers, suggesting cytotoxic T cell activation; however, EBOV-specific activation was not studied [35].

In this study, we demonstrated for the first time, to our knowledge, EBOV-specific cellular responses in seropositive asymptomatic individuals as well as EVD survivors. The seropositive asymptomatic individuals mounted stronger IFN-γ and TNF-α responses to all three LFn-EBOV fusion proteins (LFn-EBOV-NP, -VP40, and -GP1) compared to the EVD survivors. Cellular responses were significantly stronger for IFN-γ responses to LFn-EBOV-NP and LFn-EBOV-GP1 and for TNF-α responses LFn-EBOV-NP and LFn-EBOV-VP40. Consistent with previous studies, cellular responses directed to the EBOV NP were strongest compared to other EBOV antigens, in both the EVD survivors and the seropositive asymptomatic individuals. We also showed that seropositive asymptomatic individuals have IFN-γ and TNF-α cellular responses that were stronger when compared to the survivors. These results suggest that T cell immunity may play a protective role against severe EVD. Additionally, we detected EBOV-specific antibodies in serum collected from individuals living in surrounding areas of the 1976 EVD epidemic in the DRC. These results further support undiagnosed EBOV infection in individuals living in EBOV endemic regions.

While the study of long recovered SUDV survivors was unable to elicit CD8+ T cells, our study demonstrates robust CD8+ T cells against the LFn-EBOV fusion proteins in 3-year post-infection EVD survivors and 2 contacts not known to have ever been infected by EBOV. The LFn-EBOV fusion proteins elicit specific and sensitive T cell responses with low background signals in ELISPOT. Thus, LFn-EBOV priming systems may offer utility as an alternative and inexpensive technology for ex vivo screening of EBOV-specific T cell responses in future vaccine clinical trials. Additionally, our detection of both EBOV-specific CD8+ and CD4+ T cell responses suggests that the LFn delivery system is capable of efficiently presenting exogenous EBOV proteins to the MHC class I and II pathways; therefore, the use of LFn-EBOV fusion constructs presents an attractive technology for EBOV antigen delivery in vaccine design.

Our study has several limitations. Our study population is small and we recruited only a limited number of individuals with documented close EVD contact. It is likely that there are more individuals who were infected with EBOV and remained healthy during the outbreak in Nigeria. While we used different tests to identify individuals with markers of EBOV infection, we did not perform microneutralization assays to confirm infection specificity or define antiviral function of the antibodies detected in either the EVD survivors or the seropositive asymptomatic individuals. Future studies are expected to examine the functional characteristics of antibodies in minimally symptomatic and asymptomatic EBOV infections. We also demonstrated that the seropositive asymptomatic individuals have stronger T cell responses compared to the EVD survivors; however, additional studies with more study participants are needed to validate these results. We cannot rule out the possibility that the asymptomatic individuals were exposed to a low inoculum or dead antigen, precluding the development of EVD, and generated EBOV-specific T cell responses. Whether these CD8+ and CD4+ cells remain functional during secondary exposure to EBOV remains to be elucidated. Future studies investigating T cell functional markers such as PD-1 and CTLA-4 among the seropositive asymptomatic individuals are expected.

In conclusion, our findings raise new questions and highlight the need for further investigation to better understand the immune responses associated with minimally symptomatic and asymptomatic EBOV infections. By development of the LFn-EBOV ELISPOT assay, we detected cellular immune responses in EVD survivors and individuals with documented close contact with EVD patients, approximately three years after the outbreak in Lagos, Nigeria. These results suggest the importance of T cell responses in disease progression and have important implications for vaccine development, as well as for potential EBOV diagnostics based on T cell responses.

## Supporting information

S1 Checklist STROBE checklist. (DOC)

S1 Table List and characteristics of participants used in this study. (XLSX)
